# Peroxide of Hydrogen as a Medicinal Agent

**Published:** 1885-06

**Authors:** Boerne Bettman

**Affiliations:** 113 Adams St.; Chicago


ARTICLE II.

### PEROXIDE OF HYDROGEN AS A MEDICINAL AGENT.

*By* BOERNE BETTMAN, M. D., *of Chicago.*

(Read before the Chicago Society of Ophthalmology and Otology, December, 1884.)

During the last decade great advances have been made in aural therapeutics. The old remedies heretofore employed have in a great measure been substituted by others, whose introduction was actuated by the revolutionary antiseptic measures pervading all branches of medicine. Aurists were not behind hand in applying the principles expounded by Lister. Carbolic acid found its way into the recesses of the aural cavity to the detriment of the mighty horde of bacilli. The alarm had been sounded, and the watchword, let no guilty one escape, was proclaimed over the land.

The first step towards reform, towards rational medication, had been instituted, forming an epoch in the history of otology. It was soon discovered that carbolic acid, although in very many cases highly efficient in checking purulent discharges, could not be employed as a germicide; a solution possessing such properties (15%) being too strong, and not tolerated by the sensitive mucous membrane.

The boracic acid treatment proposed by Bazold, therefore, immediately found many advocates. So pronounced and rapid are the effects of this method, and so numerous the good results it has effected, that we are almost willing to promise a cure in every case of *Otitis Media Purulenta.* The bichloride of mercury has also sustained well the severe test to which it has been subjected, and may be classified among the list of remedies with which happy results may be obtained in purulent discharges from the ear. Iodoform also belongs to this category.

I desire this evening to call your attention to another equally efficient remedy, which, in my hands, has proved of the greatest value in the treatment of diseases of the ear, characterized by a purulent or muco-purulent discharge. Peroxide of hydrogen, discovered by Thenard in 1818, for many years known only to the professional chemist, was introduced into medical literature by Dr. B. W. Richardson, 1855. It was for a time extensively used, then fell into oblivion, and only lately reclaimed to the medical world by the unceasing labors of Dr. Coffin, and especially Dr. W. A. Harlan, of this city.

It has been principally employed by dentists for the treatment of alveolar abscesses. With the exception of two cases, reported by Dr. Prince, and those recorded by Dr. LeRoy Walker, I have found no mention of it in American aural or ophthalmic literature. It has been employed in the clinics of DeWecker, Landolt and other Parisian oculists. It is a highly prized antiseptic, and as such of inestimable value as a local agent.

The usual method of preparing the pure peroxide of hydrogen* is by decomposing peroxide of barium with hydrochloric acid. The chemical reaction is represented by the following equation: $Ba\ O_2 + 2\ H\ Cl = H_2\ O_2 + Ba\ Cl_2$. The chloride of barium is removed from the solution by the addition of sulphate of silver, which precipitates the barium as sulphate of baryta, and the silver as chloride of silver, thus:

$Ba\ Cl_2 + Ag_2\ O\ SO_3 = 2\ Ag\ Cl + Ba\ O\ SO_3$. The precipitates are allowed to subside, and the clear liquid evaporated in the exhausted receiver of the air-pump over a dish of oil of vitriol to absorb the water, which evaporates much more rapidly than the peroxide.

In its pure state it is a colorless sirupy liquid of a slightly bitter taste, and possesses a faint odor of chlorine. Its sp. gr. is 1.455. It is a highly unstable compound, decomposing rapidly when exposed to air and liberating its oxygen. By virtue of this property it owes its value as a therapeutic agent. The dilute substance, as sold for medicinal purposes, is more stable, and may be heated to a high temperature without losing any of its properties.

*The following table prepared by M. Miquel, of the Observatoire de Montsouris, shows the comparative value of the various antiseptics. The minimum quantity of the compound is given which will prevent the formation of germs in 1000 c. c. of beef tea:

---

* Bloxam's Chemistry, Inorganic and Organic. Page 84.

* (Bied. Centr.) Weekly Medical Review. Vol. xl., No. 1.

| | Grms. |
|---|---|
| Mercurous iodide | 0.025 |
| Silver iodide | 0.03 |
| Hydrogen peroxide | 0.05 |
| Mercuric chloride | 0.07 |
| Silver nitrate | 0.08 |
| Osmic acid | 0.15 |
| Chromic acid | 0.20 |
| Iodine | 0.25 |
| Chlorine (gaseous) | 0.25 |
| Hydrocyanic acid | 0.40 |
| Bromine | 0.60 |
| Chloroform | 0.80 |
| Copper sulphate | 0.90 |
| Salicylic acid | 1.00 |
| Benzoic acid | 1.10 |
| Potas. chromate | 1.30 |
| Picric acid | 1.30 |
| Lead chloride | 2.10 |
| Mineral acids | 2.00–3.00 |
| Essence bitter almonds | 3.20 |
| Phenol | 3.20 |
| Potas. permanganate | 3.50 |
| Aniline | 4.00 |
| Alum | 4.50 |
| Tannin | 4.80 |
| Arsenious acid | 6.00 |
| Boracic acid | 7.50 |
| Chloral hydrate | 9.00 |
| Ferrous sulphate | 11.00 |
| Amyl alcohol | 14.00 |
| Ethyl sulphide | 22.00 |
| Borax | 70.00 |
| Ethyl alcohol | 95.00 |
| Potas. thiocyanate | 120.00 |
| Potas. iodide | 140.00 |
| Potas. cyanide | 185.00 |
| Sodium thiosulphate | 275.00 |

I have made quite a number of observations with the microscope regarding the action of peroxide of hydrogen upon pus. If several drops of pus taken from a discharging ear be placed upon a slide and a drop of dioxide of hydrogen be allowed to flow under the covering glass the following phenomena will be noticed :

The pus corpuscles and bacteria are put into lively motion, small bubbles of gas, the liberated nascent oxygen, are now evolved. The pus corpuscles gradually lose their spherical form, shrink, usually assuming a crescentic form, and are heaped up a mass of detritus. The bacilli are affected in a similar manner. In a few seconds the active bodies are transformed into a dead mass, intermixed with the decomposed pus corpuscles and surrounded by seething bubbles of gas.

Whether the bacteria are totally destroyed never to be resuscitated can only be determined by a series of experiments, (attempts at cultivation) which I reserve for some future occasion; although pure oxygen is known to kill bacteria outright, which in all probability will be verified by the experiments just alluded to.

Its therapeutical action in treatment of alveolar abscess has been explained by Harlan, as follows:

*"When the peroxide of hydrogen comes into contact with pus the extra O it contains is liberated so rapidly that the hydrogen and sulphur of the tissues immediately combine, resulting in $H_2 SO_4$, or sulphuric acid in small quantity, sufficient to glaze the surface of the pus producing area, thus affording an opportunity for the exuding protoplasmic material to organize into new tissue. The remaining unsatisfied atoms of O quickly distend the pus sac and force the contents through the root or fistulous opening."

I have employed the remedy during the last six months in more than thirty cases of *Otitis Media Purulenta.* In every instance, my most sanguine expectations have been realized. The preparation employed is that sold only by chemists, containing twelve volumes of gas. I first thoroughly cleanse the ear with warm water, dry the organ by repeated introductions of

*Transactions of the American Dental Association, 1884, page 40.

absorbent cotton and then instil 8 to 12 drops of the oxygenated water. Contact with the pus and diseased tissues sets free the oxygen, visible as gas bubbles, which united with the expelled pus forms a seething, frothy mass, forcing its way outwards from its narrow confines. This decomposition goes on for several minutes. The patients rarely if ever complain of pain. They invariably speak of a boiling or effervescence in the ear accompanied by a sensation of heat, following immediately upon the introduction of the remedy. After cleaning the parts, the mucous membrane of the middle ear is seen to have assumed a very faint milky-white appearance. Repeated instillations are accompanied by less effervescence, until at last it remains in the ear as a perfectly clear liquid, no longer cognizable by the patient by the evolution of gas. I have also been informed by many individuals, that as the discharge decreased, the burning sensations produced by the remedy increased in severity. The strength of the remedy may be weakened by the addition of water.

The liberated gas enters the most hidden and remote recesses of the middle ear, forcibly dislodging the decomposing material and destroying bacteria embedded in the tissue meshes. If the perforation of the *Membrana Tympani* is small, the dioxide can be injected directly into the middle ear.

I have also employed this remedy successfully in cases of chronic dacryocystitis, and in one case of trachoma which had run its course, but where there still existed a mucid discharge. I cannot do better than relate the clinical history of one of these cases to demonstrate the curative effects of the medicine.

Miss Y. sought my advice for a chronic dacryocystitis. There was an extensive swelling of the sac, which had existed for several years. On pressure, a large quantity of muco-purulent matter was expelled into the conjunctival sac.

Introduction of a Bowman's probe revealed the presence of a tight stricture, which was forced under the influence of ether. The usual routine medication, with astringents, carbolic acid, introduction of gelatine bougies and probes, ameliorated the difficulty; but although continued for a period of six months, was not able to completely check the discharge. I therefore determined to try the peroxide of hydrogen. The very first injection was followed by a decided improvement; the discharge ceased entirely on the third application. Continued introduction of the dioxide was not accompanied by effervescence, proving conclusively the absence of inflammatory products. Contact of this medical agent with the eye produces a sharp, stinging pain, which is rapidly relieved by applications of cold water. I have thus far obtained perfect cures in four cases of dacryocystitis of long standing.

Another case still under treatment has been greatly improved and will soon be dismissed.

The preparation of peroxide of hydrogen employed in the above cases was obtained from Mr. Sargent, 125 State street. It was marked chemically pure, and proved to be so, testing it with blue litmus paper. The finer chemical tests might reveal the presence of a trace of free acid, but of so minute a quantity as to form no factor in the action of the remedy.

113 Adams St.

